# Timing of posterior capsular rupture during cataract surgery

**DOI:** 10.1038/s41433-025-03942-8

**Published:** 2025-07-24

**Authors:** Akshay Narayan, Yan Ning Neo, David Lockington, Alexander C. Day

**Affiliations:** 1 Stoke Mandeville Hospital, Aylesbury, UK; 2 Moorfields Eye Hospital NHS Foundation Trust, London, UK; 3 Whipps Cross University Hospital, Barts Health NHS Trust, London, UK; 4 Tennent Institute of Ophthalmology, Gartnavel General Hospital, 1053 Great Western Road, Glasgow, UK

**Keywords:** Outcomes research, Eye diseases

## Abstract

**Background/Objectives:**

The main aim of this study was to describe the stage at which posterior capsular rupture (PCR) occurred during cataract surgery and the association with anterior capsular (AC) tears.

**Subjects/Methods:**

This was a retrospective, observational cross-sectional study of all cataract surgeries conducted in Moorfields Eye Hospital complicated by PCR, between January 2014 and December 2018. The primary outcome measure was the stage of cataract surgery in which PCR occurred. The secondary outcome measures were the incidence of AC tears, intraoperative complications and location of intraocular lens (IOL) implantation.

**Results:**

1042 eyes were included in the study and PCR occurred at the following stages: hydrodissection in 40 (3%) eyes, phacoemsulsification in 621 (60%) eyes, irrigation-aspiration (IA) in 248 (24%) eyes, lens implantation in 87(8%) eyes, viscoelastic removal in 44(4%) eyes and wound hydration in 2 (1%) eyes. 624/1042(60%) patients had a sulcus lens, 117/1042(11%) lens in the bag, 190/1042(19%) sulcus optic capture, 24/1042(2%) AC lens and 87/1042(8%) aphakic. 15% of PCR cases were associated with an AC tear. There was a significant difference in the timing of PCR and location of IOL implantation between the eyes with an intact anterior capsulorhexis and those with an AC tear (*p* < 0.01).

**Conclusion:**

PCR is most likely to occur during the phacoemulsification stage of cataract surgery. However, surgeons should be aware that the presence of AC tears may alter this and possibly influence the location of IOL implantation.

## Introduction

Cataract surgery is the most commonly performed intraocular surgery in the world [1]. Advancements in technology, surgical techniques and anaesthesia have led to the establishment of modern small-incision phacoemulsification surgery [2, 3]. Although a very safe procedure yielding significant improvements in patients’ vision, complications may still occur [4].

Posterior capsular rupture (PCR) is the most common serious intraocular complication that can occur during cataract surgery, which is associated with an increased risk of visual loss [5]. This is because it increases the risk of the following post-operative complications: cystoid macular oedema by 2.6 times, endophthalmitis by 21 times and retinal detachment by up to 40 times [6, 7]. As the presence of such intraoperative and postoperative complications can have an adverse impact on visual outcomes following surgery, risk factors for PCR have been carefully studied and identified. It is estimated that PCR occurs in 0.9% of cataract surgeries done in the National Health Service [8]. Ocular risk factors that can increase the risk of a PCR include dense/mature/brunescent cataract, phacodenesis and pseudoexfoliation [9–11]. Extraocular factors associated with a higher risk of PCR include advanced age, inability to lie flat and trainee surgeons [12–14].

Although the ocular and systemic risk factors for PCR during cataract surgery have been established, there is limited literature on the timing of PCR during cataract surgery [15–17]. The main objective of this study was to identify the stage of cataract surgery during which PCR occurred. We also report on patient characteristics, incidence of other intraoperative complications, choice of IOL and location of IOL implantation.

## Materials and Methods

A retrospective cohort study of all cataract surgeries complicated by PCR performed at Moorfields Eye Hospital NHS Foundation Trust over a period of five years between January 2014 and December 2018, was carried out. Moorfields Eye Hospital is one of the largest providers of ophthalmic services in Europe. Through ten regional clinical commissioning groups, it caters to a population of approximately 3.7 million people within London, United Kingdom. Given that this was classified as a retrospective audit and involved analysis of data that was routinely collected by the Trust, it was exempted from institutional review board approval by the institution’s Research and Development Department.

Data was exported from the electronic medical records system employed by Moorfields Eye Hospital NHS Foundation Trust, OpenEyes™ (Apperta Foundation, England). Specific search terms were used to screen all patient letters and patient diagnoses. The search terms used were “PCR”, “posterior capsule rupture” and “posterior capsular rupture” and the medical records of patients identified through these search terms were reviewed. Recording of intraoperative complications is a mandatory field in the electronic medical records system. The operation notes and text box comments were also reviewed to identify details of intraoperative complications. We included all consecutive eyes with a PCR that occurred during routine cataract surgery in the study period. Phacoemulsification was performed using the Alcon Infiniti or Centurion Vision Systems in all cases. Subsequently, irrigation/aspiration (IA) was performed bimanually with metallic IA tips in the majority of cases. The bimanual aspiration cannula that was utilized was a curved 23-gauge cannula with a 0.4 mm top aspiration port. The bimanual irrigation cannula that was used was a curved 21-gauge cannula with 0.51 mm open-end irrigation port. Eyes that had PCR during combined corneal, glaucoma surgery or phacovitrectomy were excluded. Eyes in which there was lack of clear documentation regarding which stage of cataract surgery the PCR occurred in were also excluded. Baseline demographics, pre-operative risk factors (lack of fundal view and presence of posterior plaque), choice of IOL, location of IOL implantation, stage of PCR and presence of other intra-operative complications (anterior capsular tear, vitreous loss, zonular dialysis, dropped lens fragment) were collected for all patients.

Cataract surgery was broadly classified into six stages: hydrodissection, phacoemulsification, IA, IOL implantation, removal of viscoelastic and hydration of wounds. Patient demographics were described using descriptive statistics. Categorical variables were reported using numbers and percentages and continuous variables were reported using mean and standard deviation. Pearson’s χ^2^ test was used to analyse associations between categorical variables. A p-value of less than 0.05 was considered to be statistically significant. Statistical analyses were performed using R version 4.1.0 (R Foundation for Statistical Computing).

## Results

Over the five-year period between January 2014 and December 2018, a total of 1042 eyes with PCR during cataract surgery were included. The average age at the time of surgery was 70.3 years and 522 (50.1%) eyes were female (Table 1). There was an even spread of PCR across the five-year period and the year with the highest incidence was 2016 with 235 (23%) eyes reported. The IOL that was most frequently implanted during cataract surgery was the MA60AC lens with it being implanted in 814 (79%) eyes. The next most frequently implanted lens was the SN60WF lens in 111 (10%) eyes. 87 (8%) eyes were left aphakic following surgery with no lens being implanted. In 24 (2%) eyes, the IOL implanted was the MTA4U0 lens. The SA60AT lens was only used in 13 (1%) eyes.

**Table 1:** Patient demographics and baseline clinical parameters.

Parameter	Number of eyes, *N* = 1042 eyes
Age at time of surgery, years	70.3 ± 12.3
Sex, female:male	522 : 520
Year, *n* (%)	
2014	207 (20%)
2015	200 (19%)
2016	235 (23%)
2017	210 (20%)
2018	190 (18%)
IOL choice, *n* (%)	
MA60AC	814 (79%)
MTA4U0	24 (2%)
Null	87 (8%)
SA60AT	6 (1%)
SN60WF	111 (10%)

Anterior capsular (AC) tear was reported alongside PCR in 155 (15%) eyes (Table 2). Vitreous loss occurred in 835 (80%) eyes and no vitreous loss was observed in 207 (20%) eyes. Zonular dialysis was only observed in 47 (5%) eyes with PCR. The main location of implantation of the IOL, MA60AC, was in the sulcus and was performed in 624 (60%) eyes. In 24 (2%) eyes, the MTA4U0 lens was implanted in the anterior chamber.

**Table 2:** Intraoperative complications and location of IOL implantation.

Parameter	Number of eyes, *N* (%)
Intraoperative complication associated with PCR	
AC tear	155 (15%)
Vitreous loss	835 (80%)
Zonular dialysis	47 (5%)
Dropped nucleus	123 (12%)
Location of IOL implantation	
Sulcus	624 (60%)
In the bag	117 (11%)
Anterior chamber	24 (2%)
Sulcus optic capture	190 (19%)
Aphakic	87 (8%)

The stage during which the highest proportion of eyes experienced PCR was phacoemulsification with 621 (59.6%) eyes (Table 3). After phacoemulsification, PCR was most likely to occur during the IA stage, with 248 (23.8%) eyes having it then. During the hydrodissection and lens implantation stage, 40 (3.8%) and 87 (8.3%) eyes experienced PCR, respectively. Following IOL implantation, PCR was noted in 44 (4.2%) eyes during removal of the viscoelastic. 2 (0.3%) eyes had a PCR during the final stage of cataract surgery, wound hydration.

**Table 3:** Stage of cataract surgery during which the posterior capsular rupture occurred.

Stage	Number of eyes, *N* (%)
Hydrodissection	40 (3%)
Phacoemulsification	621 (60%)
IA	248 (24%)
IOL implantation	87 (8%)
Removal of viscoelastic	44 (4%)
Hydration of wounds	2 (1%)

Of the 1042 eyes, 155 eyes had both an AC tear and PCR. When comparing those with and without an AC tear, there was no significant difference in the age at the time of event or sex (Table 4). There was significant difference in the stage of cataract surgery during which PCR occurred between the two groups (*p* < 0.01). There was also a statistically significant difference in the location of implantation in those with AC tear compared to those without AC tear (*p* < 0.01).

**Table 4:** Comparison of the stage at which PCR occurred during cataract surgery and location of IOL implantation between the AC tear and non-AC tear groups.

	Presence of AC tear, *N* = 155 eyes	Intact anterior capsulorhexis, *N* = 887 eyes	*p*-value
Age, years	70.8 ± 14.6	69.9 ± 13.1	0.65
Sex, female:male	80:75	442:445	0.82
Stage of PCR, n			<0.01
Hydrodissection	13 (8.4%)	27 (3.0%)	
Phaco	110 (71.0%)	510 (57.5%)	
I/A	25 (16.1%)	224 (25.3%)	
IOL insertion	5 (3.2%)	82 (9.2%)	
Removal of viscoelastic	2 (1.3%)	42 (4.7%)	
Wound hydration	0 (0%)	2 (0.2%)	
Location of IOL implantation, n			<0.01
Sulcus	106 (68.4%)	518 (58.4%)	
In the Bag	7 (4.5%)	110 (12.4%)	
Anterior chamber	6 (3.9%)	18 (2.0%)	
Sulcus optic capture	6 (3.9%)	184 (20.7%)	
Aphakic	30 (19.4%)	57 (6.4%)	

## Discussion

In this consecutive series of 1042 eyes, PCR was most likely to occur during the phacoemulsification stage, followed by IA. Alongside PCR, vitreous loss was the most frequently observed intraoperative complication in these eyes. The IOL was most likely to be implanted in the ciliary sulcus in cataract surgery complicated with PCR. The presence of an AC tear influences the timing of PCR and the location of IOL implantation. In eyes with an AC tear, PCR was more likely to occur during the earlier stages of cataract surgery, such as hydrodissection and phacoemulsification. The IOL was also less likely to be implanted to the bag and placed in alternative location,s such as the anterior chamber in these eyes compared to those with an intact anterior capsulotomy.

There is very limited published data on the stage at which PCR occurs, and our finding that PCR occurred most frequently during the phacoemulsification stage of cataract surgery (60%) is consistent with that previously published, whereby PCR during phacoemulsification occurred in 41% to 57% of cases [18–20]. There are several mechanisms that increase the risk of PCR during phacoemulsification. An AC tear caused by iatrogenic trauma to the rhexis edge by the phacoemulsification probe may extend posteriorly [21]. There may also be mechanical trauma to the posterior capsule if the groove is deeper than intended [22]. Towards the end of phacoemulsification, removal of the last nuclear fragment is also another risky stage of cataract surgery for posterior capsular rupture [18].

Several studies demonstrated that PCR was most likely to occur during the IA stage of cataract surgery. Gimbel reported that PCR occurred in 53% of cases during the removal of cortical material [15]. Zheng and Lin identified that PCR occurred most frequently during the same stage in 39% of eyes in their series [16]. In our study, PCR occurred during removal of cortical material in 24% of cases, making it the stage with the second highest frequency of PCR after phacoemulsification. There are several mechanisms that could contribute to a PCR during the IA stage. Firstly, it could be attributed to the use of excessive aspiration [23]. Secondly, placement of the aspiration port close to the posterior capsule may also precipitate rupture. Finally, overzealous polishing of the thin posterior capsule may also lead to its rupture [23].

Furthermore, improvements in cataract surgery technology and instrumentation may also account for this change in the timing of PCR. In Gimbel and Zheng’s case series, IA was performed co-axially. In our study, a bimanual approach was performed for aspiration of the cortex in the majority of cases. Bimanual IA has several advantages compared to the co-axial approach. Firstly, there is greater anterior chamber stability as the instruments are inserted via the smaller paracentesis instead of the larger main incision [24]. Secondly, the surgeon has superior access to the cortex, especially the sub-incisional cortex, when IA is performed bimanually instead of co-axially. Both of these help to reduce the risk of PCR during IA. In more recent years, the adoption of silicone tips has helped to further decrease the risk of PCR during this stage. Thorough assessment and examination of the inner bore, lumen and opening aperture of metallic aspiration tips revealed that hidden, sharp irregularities are present within these tips [25]. Contact between these tips and the posterior capsule, even if minimal, could easily result in a PCR. Silicone tips help to overcome this by increasing the distance between the posterior capsule and bare metal.

As a result of a PCR, another important consideration facing the surgeon is the location of IOL implantation. Our results show that the IOL was most frequently implanted within the ciliary sulcus with the IOL placed within the sulcus in 60% of eyes. Comparatively, the IOL was implanted within the bag in only 11% of eyes. When deciding between placing the IOL in the ciliary or sulcus or in the bag, the main characteristics to consider are the size and nature of the posterior capsular tears. If the tear measures less than 6 mm and has well-defined borders not indicative of vitreous loss, the IOL may be implanted within the bag [26]. If the posterior capsular tears are larger and borders are less defined, the ciliary sulcus may be a more appropriate place to implant the IOL. Although the size and nature of the tears were not described in all the cases included in our study, Osher and Cionni found that only 10% of posterior capsular tears had well-defined margins [16]. As most posterior capsular tears may have margins that are not visible, implantation of the IOL in the ciliary sulcus may be preferable to the bag when a PCR occurs. In addition to placing the IOL within the sulcus, some surgeons may opt for sulcus placement with optic capture, and this was performed in 19% of cases in our study. This is achieved by placing the haptics of the IOL within the ciliary sulcus and the optic pushed posteriorly and secured behind a well-centred anterior capsulotomy. The main advantages of this technique compared to just placement within the sulcus is better stability and no IOL power adjustment [27]. 8% of eyes in our study were left aphakic and without any IOL following a posterior capsular rupture during cataract surgery. There are several reasons why surgical aphakia may occur. Firstly, if there is a dropped lens fragment into the vitreous, the surgeon may opt to leave the patient aphakic. This makes subsequent retrieval of the lens fragment from the vitreous easier, as there is no IOL obstructing it. Secondly, a patient may also be left aphakic if there is not sufficient capsular support to make implantation of the IOL within the ciliary sulcus or bag safe. This might occur as a result of lack of anterior capsular support, zonular dialysis, or widespread zonular dehiscence [28]. In such cases with inadequate capsular support, scleral-fixated, iris-fixated or anterior chamber lenses (AC-IOL) may be more appropriate. In our study, 2% of eyes had an AC-IOL implanted. In the absence of stable capsular support, placing an AC-IOL remains a viable option. However, as AC-IOLs increase the risk of glaucoma, uveitis, corneal endothelial cells loss and pseudophakic macular oedema, careful patient selection based on age, corneal clarity and presence of glaucoma is required [29–32].

PCR is an intraoperative complication that may occur with or without vitreous loss. In our study, PCR with vitreous loss was reported in 80% of eyes. This rate of vitreous loss is similar to the rate reported in the National Ophthalmology Database. Vitreous loss was identified in 79% of cataract surgeries that were complicated by a PCR [33]. When a PCR occurs, there is a breach in the barrier between the anterior and posterior segments. Disruption of the anterior hyaloid face causes the vitreous to advance forward into the capsular bag and leads to vitreous loss. The incidence of other intraoperative complications alongside PCR was reported in our study. AC tear and dropped lens fragments into the vitreous cavity occurred in 15% and 12% of eyes, respectively. Zonular dialysis was the least frequently reported intraoperative complication and only noted in 5% of eyes. Various studies comparing the incidence of intraoperative complications during cataract surgery have previously reported a similarly low incidence of zonular dialysis, ranging between 0.7% and 13% [5, 21, 33].

PCR occurred during hydrodissection or phacoemulsification in 80% of eyes with AC tears and in 61% of eyes without an AC tear. In this group, 14% of eyes had a PCR during IOL implantation, removal of viscoelastic or wound hydration. In the AC tear group, only 4.5% of eyes had a PCR during the same stages. These results seem to indicate an association between the presence of an AC tear and a PCR in the earlier stages of surgery. During hydrodissection, there is an increase in the hydrostatic pressure within the capsule [13]. There is also vigorous endocapsular manipulations during phacoemulsification. Both of these factors can lead to the peripheral extension of an AC tear and lead to a PCR. Although the presence of such AC tears may be associated with PCR during the earlier stages of cataract surgery, they may be associated with a lower incidence of PCR during the later stages of cataract surgery. Initially, it may be challenging to identify AC tears during the hydrodissection and phacoemulsification stages due to the presence of a significant amount of lens material within the bag. Once the nuclear fragments and cortical material have been removed, any AC tears may become more visible. As surgeons are likely to identify such AC tears during the later stages of cataract surgery and there is widespread understanding that AC tears increase the risk of PCR, they may exhibit greater caution when performing IOL insertion, viscoelastic removal, and wound hydration. It is estimated that PCR occurs in up to 24% of eyes with an anterior capsular tear during cataract surgery [34]. As a result of this higher level of caution and more delicate movements, the risk of PCR may be lower during the latter stages if there is an AC tear.

In our study, we also found that there was a significant difference in the location of IOL implantation between the AC tear and non-AC tear groups. In both groups, although the IOL was most frequently implanted in the ciliary sulcus, only 4.5% eyes with AC tear had IOL implanted within the bag, compared to that in 12.4% of eyes without AC tear. In the presence of a PCR, the IOL is only likely to be placed within the bag if the break in the posterior capsule tear is small and has well-defined boundaries. Since the morphology of AC tears extending to the posterior capsule is likely to be radial with poorly defined margins, fewer surgeons may have opted to implant the lens within the capsular bag if there was an AC tear. 23.2% of eyes in the AC tear groups had the IOL implanted within the anterior chamber or left aphakic. Comparatively, only 8.5% of eyes in the non-AC tear groups had the IOL implanted within the anterior chamber or left aphakic. If there is evidence of capsular support, it is preferable to implant an IOL within the bag or ciliary sulcus. In the absence of robust capsular support, it may be more prudent to look for alternative locations such as the anterior chamber or utilise scleral-fixated or iris-fixated lenses. Since the presence of both anterior and posterior tears highlights that there may be inadequate capsular support to support a lens within the bag or sulcus, more surgeons in the AC tear group elected to leave the patient aphakic or implant an AC-IOL.

Our study does have several limitations. Firstly, the classification of cataract surgery into stages is broad. For instance, phacoemulsification includes grooving, cracking and removal of nuclear segments. Further stratification of phacoemulsification into the steps in detail could have yielded more specific results and precise identification of the high-risk stages of cataract surgery but this is not always possible through freetext search from our EPR. Secondly, our cohort consists of a mixture of bimanual, automated and simcoe IA. Although IA was performed bimanually in most cases, it is challenging to standardise instrument choice during cataract surgery. A small proportion of surgeons utilised simcoe or automated IA instead of a bimanual approach. Thirdly, the main aim of our study was to describe the stage of cataract surgery during which PCR occurred. Further research assessing how pre-operative risk factors, such as grade of surgeon, cataract grade and zonular pathology, correlate with the incidence of intraoperative risk factors are required in the future.

In summary, this study found that PCR was most likely to occur during the phacoemulsification stage of cataract surgery. When there is a PCR, the most likely location of intraocular lens implantation was the ciliary sulcus. PCR occurred with vitreous loss in 4 of every 5 cases. Our study also highlights that the presence of an AC tear in these cases may be associated with a higher risk of PCR during the earlier stages such as hydrodissection and phacoemulsification. Surgeons must also be aware that such deficiencies in the anterior capsulotomy are likely to have an impact on the location of intraocular lens implantation.

## Summary

### What was known before:

1. There is a lack of recent studies conducted into the timing of PCR during cataract surgery.
2. PCR has been shown to occur most frequently during the phacoemulsification or irrigation-aspiration stage of cataract surgery in case series reported in the 1980s and 1990s.
3. PCR may occur secondary to an extension of an anterior capsular tear in about 1 in 4 cases with an anterior capsular tear.

### What this study adds:

1. This is the largest case series of cataract surgeries complicated with PCR that has been reported and the first one in the 21st century specifically looking at the timing of PCR during cataract surgery.
2. PCR was most frequently reported during the phacoemulsification stage and the proportion of cases with PCR during IA has decreased, compared to previous studies published in the 1980s and 1990s. This is likely due to widespread improvements in cataract surgery technology and instrumentation.
3. The first report, to the authors’ knowledge, to report that the presence of AC-tear alongside PCR may influence the timing of PCR during cataract surgery.

## Data Availability

The datasets generated during and/or analysed during the current study are available from the corresponding author on reasonable request.
